# In vivo measurements of spinal stiffness according to a stepwise increase of axial load

**DOI:** 10.1007/s00421-021-04705-5

**Published:** 2021-05-06

**Authors:** Lea Suzanne Glaus, Léonie Hofstetter, Alexandros Guekos, Petra Schweinhardt, Jaap Swanenburg

**Affiliations:** 1 Department of Chiropractic Medicine, Integrative Spinal Research ISR, Balgrist University Hospital, Balgrist Campus, Lengghalde 5, CH-8008 Zürich, Switzerland; 2 University of Zurich, Zürich, Switzerland

**Keywords:** Stiffness, Spine, Load, Lumbar, Thoracic

## Abstract

**Background:**

The spine has a complex motor control. Its different stabilization mechanisms through passive, active, and neurological subsystems may result in spinal stiffness. To better understand lumbar spinal motor control, this study aimed to measure the effects of increasing the axial load on spinal stiffness.

**Methods:**

A total of 19 healthy young participants (mean age, 24 ± 2.1 years; 8 males and 11 females) were assessed in an upright standing position. Under different axial loads, the posterior-to-anterior spinal stiffness of the thoracic and lumbar spine was measured. Loads were 0%, 10%, 45%, and 80% of the participant’s body weight.

**Results:**

Data were normally distributed and showed excellent reliability. A repeated-measures analysis of variance with a Greenhouse–Geisser correction showed an effect of the loading condition on the mean spinal stiffness [F (2.6, 744) = 3.456, *p* < 0.001]. Vertebrae and loading had no interaction [F (2.6, 741) = 0.656, *p* = 0.559]. Post hoc tests using Bonferroni correction revealed no changes with 10% loading (*p* = 1.000), and with every additional step of loading, spinal stiffness decreased: 0% or 10–45% loading (*p* < 0.001), 0% or 10–80% loading (*p* < 0.001), and 45–80% (*p* < 0.001).

**Conclusion:**

We conclude that a load of ≥ 45% of the participant’s body weight can lead to changes in the spinal motor control. An axial load of 10% showed no significant changes. Rehabilitation should include high-axial-load exercise if needed in everyday living.

## Introduction

A well-working spinal motor control system is needed to protect the human spine from injury and to prevent low back pain (van Dieen et al. 2019; Shumway-Cook and Woollacott 2012). During both static and dynamic movements, different motor control subsystems guarantee spinal stabilization (Panjabi 1992a; Cholewicki et al. 2000). A passive subsystem is based on the biomechanical properties of the vertebrae, facet joints, spinal disks, ligaments, and joint capsules. It mainly assures end-range motion stability (Arjmand and Shirazi-Adl 2005). An active subsystem is a muscle system that reacts with altered muscle tension and muscle activity to force vector changes in relation to the spine (Bergmark 1989). The neurological subsystem receives information from passive and active subsystems regarding the position and motion of the spine. The neurological subsystem determines the spinal stability status and acts on requirements to continuously stabilize the spine (Ritzmann et al. 2015; Frank et al. 2013). For the active subsystem, muscle activity is typically used as a proxy measure (Needle et al. 2014; Shumway-Cook and Woollacott 2012). In contrast, the passive subsystem has been examined with studies that use in vitro human samples or in vitro porcine models (Stokes and Gardner-Morse 2003; Gardner-Morse and Stokes 2003; Zhang et al. 2020). Such in vitro studies test the passive structures, including bones and ligaments, but they do not include muscle activity or motor control of the spine (Stokes and Gardner-Morse 2003; Gardner-Morse and Stokes 2003; Zhang et al. 2020). Both the active and passive subsystems can be assessed by measuring spinal stiffness (Hausler et al. 2020; Swanenburg et al. 2018, 2020). Spinal stiffness can be seen as a proxy for the resistance to deformation of all subsystems together in vivo (muscles, joints, and ligaments) to the energy infused by the impulse. A device generates an impulse, which is applied to the spinous process in posterior–anterior direction (Swanenburg et al. 2018). The energy introduced by the impulse produces a reaction from muscles, joints, and ligaments; the impulse response. This impulse response is therefore a practical in vivo measure of the stiffness of the spine (Leach et al. 2003).

Spinal stiffness has been observed to decrease in response to hypergravity conditions (1.8 g) induced by parabolic flights (Swanenburg et al. 2018, 2020). These studies observed an increase in lumbar flexor and extensor muscle activity, and the decrease in lumbar curvature during hypergravity, which lead to the interpretation that the load shifts from the spine to the pelvis and thorax with the increased axial pressure during hypergravity (Swanenburg et al. 2018, 2020; Bergmark 1989). This finding was confirmed in a study with 100 healthy young participants, which showed significantly decreased spinal stiffness during standing with an additional axial load of 50% of the participant’s body weight (Hausler et al. 2020). This is in contrast to studies that measured only the passive subsystem that observed an increase in spinal stiffness and additional loading (Hausler et al. 2020). This discrepancy might be explained by the small contribution of the passive structures to spinal stability (Hodges et al. 2013) and relatively larger changes in the active subsystem.

Despite the previous findings regarding the in vivo spinal stiffness decreases with one large axial load or hypergravity (Hausler et al. 2020; Swanenburg et al. 2020, the behavior of spinal motor control with smaller levels of additional loads remains to be elucidated.

We therefore designed the present study to evaluate the effects of varying magnitudes of additional axial loads on in vivo thoracic and lumbar spine stiffness. We hypothesized that spinal motor control behaves differently with different additional axial load magnitudes. A stepwise increase of additional axial loads up to 80% of the participant’s body weight was used in a within-subject design.

## Methods

### Participants

Healthy young participants aged between 18 and 30 years were recruited for this study by word-of-mouth. All participants provided written informed consent. Participants were excluded if they had acute or chronic back pain, history of radiating pain down the leg, previous thoracic or lumbar spine surgery, spinal tumors, local infection, or any spinal fractures. The sample size was determined based on a previous study that measured lumbar and thoracic spinal stiffness with and without an additional axial load of 50% of the participant’s body weight (Hausler et al. 2020). In that study, the mean spinal stiffness values with and without axial loading were 46.6 ± 4.6 and 49.8 ± 4.2 N (Hausler et al. 2020). We used the g*power software to calculate the sample size needed (Faul et al. 2007). The minimum required sample size for the present study was 14 with an alpha level of 0.05 and a power of 0.8 and medium effect size of 0.72, which we estimated based on a previous mentioned study (Hausler et al. 2020). We follow the recommendations to consider effect sizes of 0.2 as small, 0.5 as medium, and 0.8 as large (Cohen 1988). Measurements were performed at Balgrist University Hospital, Zurich, Switzerland. This study was approved by the ethics committee of the Canton of Zurich (BASEC-Nr: 2017–01,245), and it is registered at ClinicalTrials.gov (Identifier: NCT03495843).

### Data collection

Demographic data included weight, sex, age, and height. To prevent location bias, the spinous process of L5 was identified using a portable ultrasound device (Aloka SSD-500 with an Aloka UST-934 N-3.5 Electronic Convex Probe; Aloka Co., Tokyo, Japan). The remaining lumbar and thoracic spinous processes were located by counting from L5 and marked with ink.

Posterior-to-anterior lumbar and thoracic spinal stiffness was assessed in one session. Before data collection, the procedure was explained to the participant, and one familiarization test measurement was performed. Thereafter, the first two measurements were performed with no axial load in a normal upright standing position. Then, two measurements with 10%, 45%, and 80% of their body weight as an additional load were conducted, resulting in eight measurements. The 10% and 80% loading were chosen based on the literature (Swanenburg et al. 2018; Eriksen et al. 1999). Additionally, the mid-point between 80 and 10%, i.e., 45% was chosen. A 2-min break between measurements was considered to regain tissue slack (viscoelasticity) (Stanton and Kawchuk 2009).

**Fig. 1:**
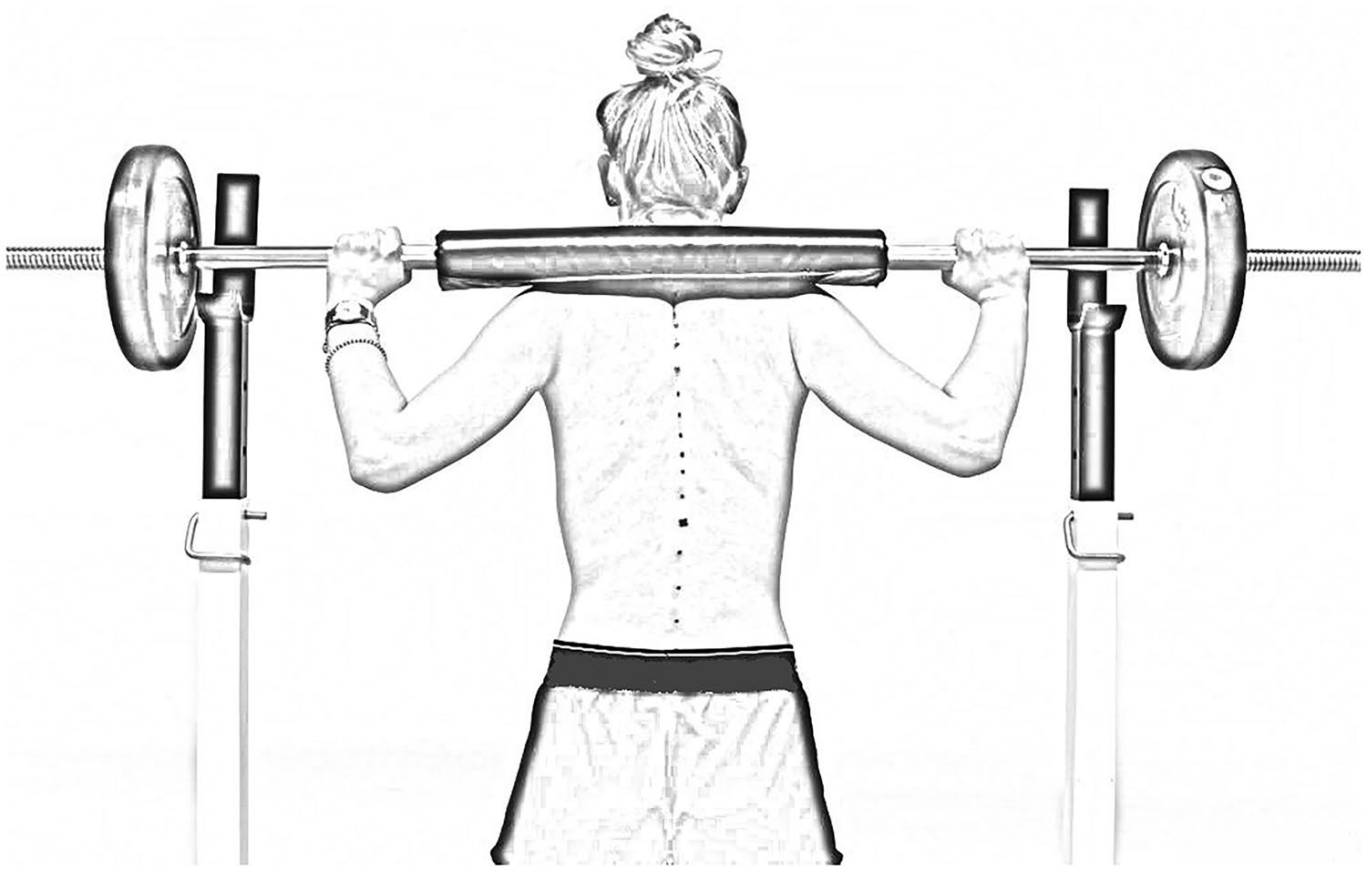
Measurement setup; standing with additional axial load

Spinal stiffness can be influenced by pain, increased abdominal pressure, and the respiratory cycle (Shirley et al. 2003; Brodeur and DelRe 1999; Hodges et al. 2005). Therefore, participants were instructed to inhale and exhale comfortably and then to hold their breath at the end of a normal exhalation. Between thoracic and lumbar spine measurements, an additional break of two breathing cycles was provided (Hausler et al. 2020). Participants were asked to report pain that they might experience during these measurements (Hausler et al. 2020). The T1 and T2 vertebrae were not measured, because the spinous processes were obstructed by the weight bar. The measurement setup is shown in Fig. 1.

### Axial load

To add an axial load, a long weight bar was used and placed on the participant’s shoulders while standing. For safety reasons, measurements were carried out at a squat rack slightly below the participant’s shoulder height. Then, participants were asked to place their feet directly under the middle part of the bar and put their hands on a predefined grip. A soft pipe insulation element was fixed around the weight bar to reduce the pressure on the participant’s shoulder. Measurements were executed when participants were standing upright in a stable position after they had picked up the bar.

### Spinal stiffness assessment

Spinal stiffness was defined as the reaction to the deformation of the spinal system given by the impulse response. The impulse response characterizes the reaction of a linear, time-invariant system to a very brief (< 1 ms) impulse (Girod et al. 2003). Because of the time-invariance, the reported units of the impulse response are Newton (instead of Newton*seconds for classical impulses). The reaction of the spinal system (bones, ligaments, disks, and muscles) to the energy infused by the impulse is thus a proxy for spinal stiffness (Leach et al. 2003; Hofstetter et al. 2018). This method can be used in the upright standing and changing axial loading situations. Posterior-to-anterior spinal stiffness was measured using a computer-assisted analytic device (PulStar Function Recording and Analysis System device PulStarFRAS; Sense Technology Inc., Pittsburgh, PA), which has good-to-excellent reliability (Leach et al. 2003; Hausler et al. 2020).

### Measurement procedures

The participant was asked to stand freely in a neutral position. The pelvis or arms were not fixed during the measurement. Spinal stiffness can be influenced by pain, increased abdominal pressure, and the respiratory cycle (Shirley et al. 2003; Brodeur and DelRe 1999; Hodges et al. 2005). Therefore, participants were instructed to inhale and exhale comfortably and then to hold their breath at the end of a normal exhalation. Between thoracic and lumbar spine measurements, an additional break of two breathing cycles was provided (Hausler et al. 2020). At a 90° angle, the impulse head was pressed with a single contact probe lightly against the participant's spinous process in the posterior–anterior direction. To compensate for possible soft-tissue components between the impulse head and spinous process and to ensure that the measurement started at the same initial point, a preload of 18 N was applied. An 80 N pulse was applied to measure stiffness. Participants were asked to inform the investigator immediately if they experienced pain during the measurement.

### Data analysis

Participant characteristics were summarized by descriptive statistics. The mean value of two measurements for each loading condition was calculated. Deviation from normal data distribution was assessed using the Shapiro–Wilk test. A repeated-measure analysis of variance (ANOVA) with spinal stiffness as a dependent variable and different loads as a within-participant factor was used. Vertebral levels (T3–L5) were used as a continuous covariate. Post hoc testing using Bonferroni correction was used. Data were collected and stored using REDCap (8.2.0, Vanderbilt University). For statistical analysis, SPSS 25 (IBM, PASW Statistics, Chicago, IL) was used.

## Results

### Participants

**Fig. 2:**
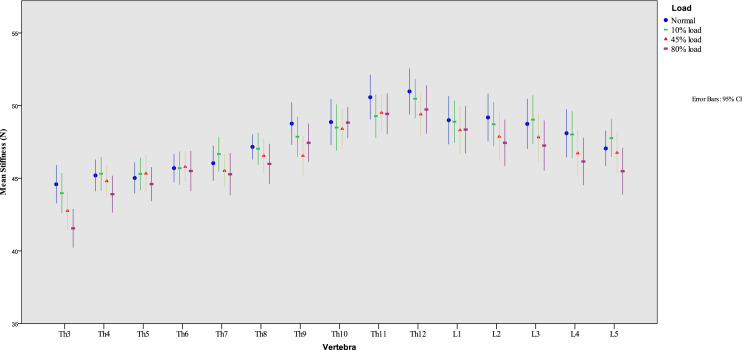
Mean values of spinal stiffness and 95% confidence intervals at each loading condition for each vertebra

A total of 19 participants were recruited (mean age, 24 ± 2.1 years; 8 males and 11 females). No participant had to be excluded, and no participant felt pain during or after any of the measurements. Table 1 lists the participant characteristics, and Fig. 2 shows the mean spinal stiffness under the different loading conditions for each vertebra.

**Table 1:** Characteristics of participants

	All (*n* = 19)	Male (*n* = 8)	Female (*n* = 11)
Age (years, mean ± SD)	24 ± 2.1	24.8 ± 1.5	23.5 ± 2.3
Weight (kg, mean ± SD	64.2 ± 9.6	71.8 ± 10.0	58.7 ± 4.1
Height (cm, mean ± SD)	170.7 ± 7.9	176.6 ± 7.6	166.5 ± 4.8
BMI < 20	3	0	3
BMI 20–24.9	15	7	8
BMI > 25	1	1	0

*SD* standard deviation, *BMI* body mass index

### Influence of additional axial loading

The Shapiro–Wilk test indicated a normal data distribution in all loading conditions (p's = 0.130 (no loading)/0.616 (10% load)/0.676 (45% load)/0.819 (80% load)). A repeated-measures ANOVA with a Greenhouse–Geisser correction showed a significant effect of loading condition on mean spinal stiffness [F (2.6, 744) = 3.456, *p* < 0.021]. No significant interaction was observed between the vertebral level and loading [F (2.6, 741) = 0.656, *p* = 0.559]. Post hoc tests using Bonferroni correction revealed that with 10% additional axial load, stiffness remained unchanged (*p* = 1.000). With every additional step of loading, spinal stiffness significantly decreased: 0%/10–45% loading (p's < 0.001), 0%/10–80% loading (p's < 0.001), and 45–80% (*p* < 0.001). All mean and standard deviation stiffness values can be found in Table 2.

**Table 2:** The mean stiffness values found in *vertebras* Th3-L5 under all axial conditions

		Normal	10% Load	45% Load	80% Load
Th3	Mean	44.58	43.98	42.76	41.56
	SD	2.73	2.85	2.78	2.76
Th4	Mean	45.19	45.31	44.81	43.91
	SD	2.26	2.42	2.21	2.62
Th5	Mean	45.02	45.30	45.33	44.60
	SD	2.21	2.31	2.67	2.43
Th6	Mean	45.70	45.70	45.79	45.50
	SD	2.04	2.38	2.15	2.88
Th7	Mean	46.04	46.67	45.52	45.27
	SD	2.51	2.47	2.34	3.02
Th8	Mean	47.16	47.03	46.55	45.99
	SD	1.78	2.29	2.52	2.86
Th9	Mean	48.76	47.86	46.55	47.44
	SD	3.02	2.85	2.80	2.72
Th10	Mean	48.87	48.49	48.41	48.83
	SD	3.29	3.31	2.83	2.24
Th11	Mean	50.57	49.27	49.51	49.43
	SD	3.18	3.13	2.61	2.91
Th12	Mean	50.97	50.47	49.40	49.73
	SD	3.29	2.80	3.02	3.43
L1	Mean	48.99	48.89	48.32	48.35
	SD	3.44	3.01	3.43	3.39
L2	Mean	49.18	48.71	47.87	47.44
	SD	3.39	3.13	3.45	3.31
L3	Mean	48.74	49.03	47.82	47.25
	SD	3.58	3.49	3.43	3.58
L4	Mean	48.09	48.01	46.73	46.15
	SD	3.43	3.36	3.24	3.38
L5	Mean	47.05	47.77	46.76	45.49
	SD	2.52	2.71	2.97	3.32

*SD* standard deviation

## Discussion

This study showed decreased spinal stiffness when the additional axial load was equal to or greater than 45% of the participants’ body weight. Conversely, no change was observed with an additional load of 10% of the body weight.

Decreased lumbar spinal stiffness with higher axial loads might be the result of the changes in the lumbar motor control strategy (Swanenburg et al. 2020). It has been previously observed that hypergravity causes a decrease in spinal stiffness, increase in lumbar muscle activity, and a flattening of the lumbar curvature (Swanenburg et al. 2020). Thus, it seems that an increased activation of the global muscle system dominates over the increased activation of the local muscle system and the flattening of the lumbar spine in hypergravity. In a study using an additional axial load of 32 kg to the upper thoracic spine, corresponding to 42% of the mean weight among all participants, an increase in recruitment and activation of abdominal muscles was observed (Cholewicki et al. 1997). The activation of the abdominal muscles leads to a load shift away from the spine and directly transfers the load to the thoracic cage and pelvis (Bergmark 1989). This results in spinal de-loading, which is expected to lead to decreased spinal stiffness. Therefore, the decreased stiffness observed for large additional loads in this study can be interpreted to reflect spinal motor control changes, specifically additional engagement of the abdominal muscles. In line with this interpretation, motor control changes of walking patterns have been observed when a person is carrying large extra loads (Martin and Nelson 1986).

In contrast to the results for additional axial loads of ≥ 45% of the participants’ body weight, no changes in stiffness were observed for the 10% additional load in this study. A study on participants carrying a backpack weighing between 15 and 30% of their body weight showed only a minimal increase in abdominal forces (Goh et al. 1998). This is in line with the findings of this study, because the unchanged spinal stiffness with 10% additional load can be interpreted as no changes in motor control strategy.

Spinal stiffness in in vivo evaluations should be noted to include measurements of all subsystems, and the measured value represents the net effect of all combined subsystems. As this makes in vivo measurements of spinal stiffness ecologically relevant, no information with respect to individual subsystems is obtained. Thus, the stiffness of the passive subsystem also possibly increased in this study, as shown in the in vitro study (Edwards et al. 1987), but the net result clearly showed decreased spinal stiffness for large additional loads and no change for 10% extra load. Therefore, a potential increase in lumbar spinal stiffness as a result of higher passive resistance appears to be negligible compared to decreased spinal stiffness from other subsystems. Thus, the results of this study support that of the previous literature, demonstrating that in a neutral position, the spine is mainly stabilized by the active subsystems, i.e., the motor control and muscle system (Swanenburg et al. 2020; Panjabi 1992b).

The results of this study of a possible change of spinal motor control might be particularly interesting from a clinical point of view. Nonfunctioning motor control of the spine can affect the alignment of the lumbar spine segments, resulting in considerable strain on the lumbar spine (van Dieen et al. 2019).

### Limitations

Participants’ arms were hanging along the torso when measuring spinal stiffness with no additional load. When the additional load was fixed, the hands were placed at the weight bar, which was laid over the shoulder. Lifting the arms leads to an increased shoulder muscle activity, which, in turn, results in increased spinal stiffness (Escamilla et al. 2009). However, this increased stiffness due to arm posture appears to be small as this study observed a net decrease in stiffness and found changes between loading conditions. This study showed significantly decreased stiffness with increasing load. The minimal delectable change found in a previous study with 50% loading was larger than the difference between loading conditions in this study. These results could be due to the measurement error, but a clear direction of stiffness was found to decrease with increased loading.

## Conclusion

This study found that a load of ≥ 45% of the participant’s body weight can lead to spinal motor control changes, whereas an axial load of 10% showed no significant changes. Rehabilitation should include high-axial-load exercise if needed in everyday living.

## Data Availability

The datasets used and/or analyzed during the current study are available from the corresponding author on reasonable request.

## Abbreviations

ANOVA: Analysis of variance
BMI: Body mass index
SD: Standard deviation
